# Cost-effectiveness analysis of the SMART quit clinic program in smokers with cardiovascular disease in Thailand

**DOI:** 10.18332/tid/161024

**Published:** 2023-04-05

**Authors:** Alyssa Grant, Chia Jie Tan, Somkiat Wattanasirichaigoon, Suthat Rungruanghiranya, Araya Thongphiew, Kednapa Thavorn, Nathorn Chaiyakunapruk

**Affiliations:** 1 School of Epidemiology and Public Health, University of Ottawa, Ottawa, Canada; 2 Ottawa Hospital Research Institute, Ottawa, Canada; 3 Department of Pharmacotherapy, College of Pharmacy, The University of Utah, Salt Lake City, United States; 4 Faculty of Medicine, Srinakharinwirot University, Bangkok, Thailand; 5 Thai Physician Alliance Against Tobacco, Bangkok, Thailand; 6 Diabetes Mellitus and Endocrine Center, Paolo PhaholyothinHospital, Bangkok, Thailand; 7 Faculty of Pharmacy, Chiang Mai University, Chiang Mai, Thailand; 8 School of Pharmacy, Monash University Malaysia, Subang Jaya, Malaysia; 9 IDEAS Center, Veterans Affairs Salt Lake City Healthcare System, Salt Lake City, United States

**Keywords:** Thailand, smoking cessation, cardiovascular disease, cost-effectiveness analysis

## Abstract

**INTRODUCTION:**

The SMART Quit Clinic Program (FAHSAI Clinic) has been implemented in Thailand since 2010; however, it remains unclear whether the benefits gained from this program justify its costs. We assessed its cost-effectiveness compared to usual care in a population of Thai smokers with cardiovascular disease (CVD) from a societal perspective.

**METHODS:**

We conducted a cost-utility analysis using a Markov model to simulate lifetime costs and quality-adjusted life years (QALYs) of Thai smokers aged ≥35 years receiving smoking cessation services offered from FAHSAI Clinic or usual care over a horizon of 50 years. The model used a 6-month continuous abstinence rate from a multicenter prospective study of 24 FAHSAI Clinics. A series of sensitivity analyses including probabilistic sensitivity analysis were conducted to assess robustness of study findings. Cost data are presented in US$ for 2020.

**RESULTS:**

The FAHSAI Clinic was dominant as it was less costly ($9537.92 vs $10964.19) and more effective (6.06 vs 5.96 QALYs) compared with usual care over the 50-year time horizon. Changes in risks of stroke and coronary heart disease among males had the largest impact on the cost-effectiveness findings. The probability that FAHSAI Clinic was cost-effective was 99.8% at a willingness-to-pay threshold of $5120.

**CONCLUSIONS:**

The FAHSAI Clinic smoking cessation program was clinically superior and cost-saving compared to usual care for Thai patients with CVD in all scenarios. A budget impact analysis is needed to estimate the financial impact of adopting this program within the Thai healthcare system.

## INTRODUCTION

Clinical and epidemiological studies have consistently linked cigarette smoking to a higher incidence of cardiovascular disease (CVD)^1^. In Thailand, the prevalence of smoking has been reported to be 19% in 2017^2^. The total number of coronary heart disease cases attributable to smoking was estimated to be 52605 cases per year, costing approximately 20859 million Thai Baht (THB 31.25 = US$1.0 for 2020)^3,4^. Cigarette smoking, as a modifiable risk factor of CVD, presents as an appealing target to reduce the morbidity and mortality associated with CVD.

Smoking cessation has been found to confer cardiovascular benefits among smokers, with an immediate drop in the incidence of thrombotic events, followed by a decline of 61% and 42% in death associated with coronary heart disease and stroke, respectively, within 5 years^5,6^. Smoking cessation has also been shown to be more cost-effective compared to other preventive cardiology measures, with incremental cost-effectiveness ratios ranging from $2000 to $6000 per life-year saved compared to $9000 to $26000 per life-year saved for the treatment of hypertension or $50000 to $196000 for the treatment of hyperlipidemia. In Thailand, cost-effectiveness analyses have previously found that a range of smoking cessation interventions, including counselling (via phone and in the hospital) and pharmacotherapy such as bupropion, nortriptyline and varenicline, are cost-saving compared to unassisted smoking cessation^7^.

The SMART Quit Clinic Program (FAHSAI Clinic), established in 2010, provides smoking cessation services in all Thai provinces through a multidisciplinary team consisting of a physician, nurse, nurse assistant, public health technician, pharmacist, Thai traditional practitioner, and dentist contingent on the workforces and resources of the settings. In settings that engage with FAHSAI Clinics, typical therapy includes behavioral counselling, pharmacological treatment, as well as the prevention, monitoring and empowerment of smokers. In Thai settings that do not engage FAHSAI Clinics or other smoking cessation clinics, the standard treatment comprises unassisted quitting or brief counselling. It remains unknown whether the benefits of this national smoking cessation program justify its investment, in particular among patients with CVD in Thailand. The objective of this study was therefore to formally evaluate the cost-effectiveness of the smoking cessation services delivered via the FAHSAI Clinic to smokers with CVD disease.

## METHODS

### Overall description

We performed a cost-utility analysis of FAHSAI Clinic compared with usual care in a population of Thai smokers with CVD disease, from a societal perspective. The effectiveness and cost of FAHSAI Clinic were primarily based on data from a multicenter prospective observational study of 24 FAHSAI clinics across 21 provinces of Thailand^8^. The cohort study included 2041 participants who were first commencing the smoking cessation program in the multidisciplinary clinics and had a mean age of 44.56 years^9^. We used an annual cycle length. As per Thai HTA guidelines^9^, we discounted QALYs and costs at 3% per year^10^. This work is reported in accordance with the Consolidated Health Economic Evaluation Reporting Standards 2022 (CHEERS 2022)^11^ (Supplementary file) and complies with the Guidelines for health technology assessment (HTA) in Thailand (Second edition)^9^.

### Model structure

We developed a Markov model to simulate lifetime costs and quality-adjusted life years (QALYs) of a hypothetical cohort of smokers receiving smoking cessation services from either FAHSAI Clinic or usual care over a lifetime horizon, i.e. 50 years, as this accounts for all costs and medically important events (including the risk of a cardiovascular event and death). Our target population included Thai men (93%) and women (7%) aged 35 years, who regularly smoke 10–20 cigarettes per day. An age of 35 years was selected as it approximates the average age of Thai smokers^12^.

After commencing the smoking cessation program or usual care, a patient in the stable CHD/stroke state could remain in this state or transition to an acute disease exacerbation state. Patients in acute disease exacerbation states could either remain in this state or transition to a state of stable secondary stroke/CHD or stable CVD. The transition to the next CVD health state is assumed to be irreversible. Patients could also transition to the states of lung cancer, COPD, mouth cancer or death at any point in the model. As patients moved through different health states cycles, they accrued direct healthcare costs, life-years, and QALYs.

**Figure 1:**
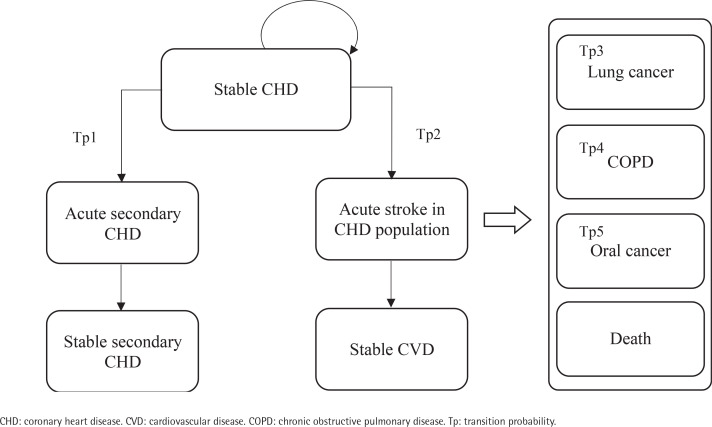
Markov model of the coronary heart disease cohort health states applied for cost-effectiveness analysis

**Figure 2:**
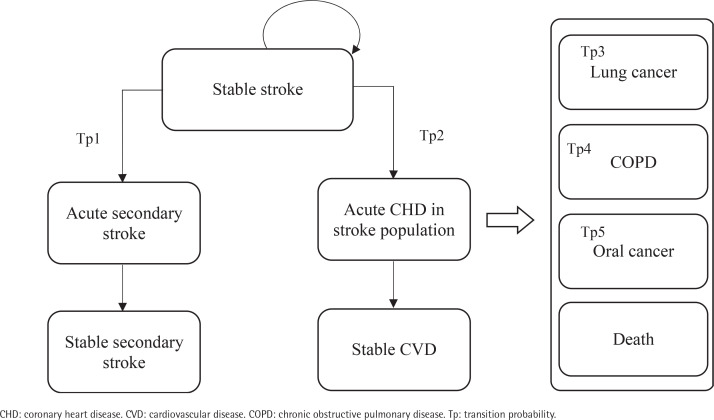
Markov model of the stroke cohort health states applied for cost-effectiveness analysis

This model has two facets representing the two cohorts of the starting population: patients with stable coronary heart disease (CHD) (Cohort A) and patients with stable stroke (Cohort B), each consisting of 9 mutually exclusive, discrete health states. The health states within the CHD cohort facet of the model include: 1) stable CHD, 2) acute secondary CHD event, 3) stable secondary CHD, 4) acute stroke in CHD population, 5) stable CVD, 6) mouth cancer, 7) lung cancer, 8) chronic obstructive pulmonary disease (COPD), and 9) death (Figure 1). The health states within the stroke cohort facet of the model are: 1) stable stroke, 2) acute secondary stroke event, 3) stable secondary stroke, 4) acute CHD in stroke population, 5) stable CVD, 6) mouth cancer, 7) lung cancer, 8) COPD; and 9) death (Figure 2). The age-stratified transition probabilities used in the CHD and stroke cohort models are presented in the Supplementary file Tables 1 and 2, respectively. In this model, there were 3 main assumptions: 1) each smoker only receives one smoking cessation therapy during their lifetime; 2) the risk of smoking related diseases was dependent on the current health state or a limited set of previous states; and 3) smokers will suffer from at most one smoking-related disease during their lifetime.

**Table 1:** Base case analysis results

*Results*	*FAHSAI clinic*	*Usual care*
Cost of treatment	$60.16	$0.00
Other medical costs	$9477.76	$10964.19
Total cost	$9537.92	$10964.19
QALYs	6.06	5.96
Incremental cost	-$1426.27	
QALY gain	0.10	
ICER	Dominant	

QALY: quality-adjusted life years. ICER: incremental cost-effectiveness ratio. FAHSAI Clinic: The SMART quit clinic program.

**Table 2:** Scenario analysis results based on effectiveness derived from a per-protocol analysis

*Results*	*FAHSAI clinic*	*Usual care*
Cost of treatment	$60.16	$0.00
Other Medical Costs	$9697.34	$10964.19
Total cost	$9757.50	$10964.19
QALYs	6.40	5.97
Incremental cost	-$1206.66	
QALY gain	0.61	
ICER	Dominant	

QALY: quality-adjusted life years. ICER: incremental cost-effectiveness ratio. FAHSAI Clinic: The SMART quit clinic program.

### Input parameters

*Transition probabilities*

Risks of developing smoking-related diseases were based on published studies. The risk of CHD among current and former smokers were derived from the Kaiser Permanente Medical Care Program cohort study, which had a mean follow-up length of 6.1 years^13^. The risk of stroke and COPD were based on CHD statistics published by the British Heart Foundation Statistics database^14^. The risk of lung cancer was obtained from a nationwide American Cancer Society prospective cohort^15^, while the risk of oral cancer was based on results of a Japanese prospective cohort^16^.

The model accounts for the reduced risk of developing smoking-related conditions among smokers who quit successfully (ex-smokers) which were compared with continuing smokers (smokers). The risks smoking-related disease and disease specific mortality were derived from targeted literature searches. Sex-specific relative risks of death from CHD, stroke, and COPD in current and former smokers were derived from the Kaiser Permanente Medical Care Program cohort study^13^. The risk of death from oropharyngeal and lung cancer by smoking status were obtained from Japanese cohort studies^16,17^. The relative risks of incident smoking-related disease were calculated by multiplying the number of Thai smokers by sex-specific absolute risks of smoking-related disease and the relative risk of disease-specific mortality and then divided by the total number of smokers. Transition probabilities from a CHD or stroke health state to death were derived by multiplying Thai age-specific mortality rates from the National Statistical Office of Thailand by the relative risks of disease specific mortality^18^.

The effectiveness of FAHSAI Clinic was based on 6-month continuous smoking abstinence rate (CAR) reported in the concurrent multicenter prospective cohort study of FAHSAI Clinics. In this study, 13.8% of participants enrolled in the FAHSAI Clinic abstained from smoking at 6 months of follow-up^8^. As FAHSAI Clinics are well established across Thailand and considered as standard care to all Thai smokers, it would not be feasible and ethical to include a control group who did not receive smoking cessation service as a comparison. We assumed that 5% of patients receiving usual care for smoking cessation abstain from smoking at 6 months of follow-up based on opinion of clinical experts (SW, SR, AT). As this quit rate follows a binary distribution, its standard error can be estimated from:


$$\frac{\text{σ}}{\sqrt{\text{n}}}=\frac{\sqrt{\text{npq}}}{\sqrt{\text{n}}}$$

where σ is a standard deviation, *n* is the number of smokers that participated in the study, and *p* is the quit rate and *q*=1-*p*.

*Cost and health utility values*

Costs were calculated from a societal perspective. In addition to smoking cessation treatment costs, other costs considered in the model encompassed other medical costs (including medication and use of health services). As per the HTA guidelines for Thailand, indirect costs were not included as we have accounted for health utilities in our model^9^. The costs for CHD and stroke in the first and subsequent years came from cost studies and expert opinion^19-22^. Direct medical and non-medical costs for COPD, oral and lung cancer were also estimated from Thai studies^21,23,24^ and data collected at the Maharajnakorn Chiang Mai Hospital and validated based on expert opinion of research team clinicians (SW, SR, AT). Cost data were estimated in Thai Baht and converted to US$ using a rate of THB 31.25 to US$1. All costs were adjusted to 2020 dollars using the Consumer Price Index^25^. Baseline utility values were derived from a cost-effectiveness analysis of Belgium smokers, an interim determination of health gain from oral cancer and precancer screening done in the UK, and from a prospective quality-of-life survey on advanced non-small-cell lung cancer patients in Europe, Canada, Australia, and Turkey^26-28^. The model input parameters are summarized in Supplementary file Table 3.

### Sensitivity analyses

We conducted a series of sensitivity analyses to assess the robustness of our results. One-way sensitivity analysis was performed for baseline event rates, relative risk estimates, costs, utility values, and the discount rate used in the model. We also conducted a probabilistic sensitivity analysis (PSA) for all parameters in the model using a Monte Carlo simulation technique with 1000 iterations, which was used to create a cost-effectiveness acceptability curve. Beta distributions were used for transition probabilities and utility values, the log-normal distribution was used for relative risk, and the gamma distribution was assigned to cost data. The PSA results were used to create cost-effectiveness acceptability curves, which show the probability of FAHSAI Clinic being cost-effective over a range of willingness-to-pay thresholds. As there were large difference in the 6-month CARs obtained in the per-protocol and intention to treat (ITT) analyses^8^, a scenario analysis was conducted to explore variation in our results that resulted from the use of effectiveness estimates measured per-protocol versus ITT.

## RESULTS

From a societal perspective, the smoking cessation program delivered by FAHSAI Clinic was associated with a lower cost ($9537.92 vs. $10964.19) and an increase in QALYs (6.06 vs 5.96 QALYs), suggesting that FAHSAI Clinic was dominant compared to usual care over a 50-year time horizon (Table 1). Results from one-way sensitivity analyses indicated that the cost-effectiveness findings were highly sensitive to changes in the relative risks of stroke among former smoking males aged 35–64 years and of CHD among male smokers (Supplementary file Figure 1). Nevertheless, FAHSAI Clinic remained cost-saving within the parameters employed in the one-way sensitivity analyses (Supplementary file Figure 2).

Probabilistic sensitivity analysis indicated that FAHSAI Clinic was cost-saving in 99.8% of 1000 simulations at a Thai willingness-to-pay value of $5120.00 (Supplementary file Figure 3). Results from the scenario analysis using the per-protocol efficacy estimates indicate that the increased efficacy value had less costs associated with gains in QALYs compared to the base-case analysis, although FAHSAI Clinic remained dominant over usual care in both scenarios (Table 2).

## DISCUSSION

This is the first study to estimate the cost-effectiveness of the multidisciplinary FAHSAI Clinic in CVD patients in Thailand. Our findings suggest that FAHSAI Clinic is likely to be cost-effective among Thai smokers with CVD compared to usual care from a societal perspective. Over a 50-year time horizon, the FAHSAI Clinic is both less costly and more effective compared to usual care, suggesting that this smoking cessation intervention is cost-effective. Our study findings were robust to changes in input parameters and model assumptions but were sensitive to the risks of developing stroke and CHD, and among males.

Our findings are consistent with previous cost-effectiveness analyses assessing other smoking cessation interventions and programs in Thailand. One study found that a structured community pharmacist-based smoking cessation program was cost-saving and more effective than usual care^29^. However, this study utilized a healthcare system perspective and therefore indirect costs (i.e. future costs from productivity loss) were not included in their analyses. Another study found that smoking cessation interventions including a combination of counseling and pharmacotherapy (varenicline or nortriptyline) were cost-effective compared with unassisted cessation^7^. Finally, a study assessing the cost-effectiveness of pharmacological therapies for smoking cessation in COPD patients in Thailand found that varenicline is the most cost-effective strategy, compared to nortriptyline and bupropion^30^. However, due to a lack of Thai data, these studies derived the effectiveness of the smoking cessation interventions from other countries, which might not be generalizable to the Thai population^7,29,30^. Our study was based on real-world evidence on the effectiveness and costs of FAHSAI Clinic and usual care in Thailand, and therefore relies on fewer assumptions than the previous cost-effectiveness studies of smoking cessation interventions.

In comparison with our findings indicating the multidisciplinary FAHSAI Clinic was cost-effective compared to usual care, a recent meta-analysis and cost-effectiveness analysis of smoking cessation interventions reported that behavioral interventions were the most cost-effective options, compared with pharmacological and combined pharmacological and behavioral interventions^31^. The studies included in the meta-analysis, however, were done in Switzerland, Denmark, Germany, and the US, all of which had different healthcare systems and smoking cessation guidelines from Thailand. As such, this greatly limits the generalizability of these findings to Thai smokers.

### Limitations

This study has some limitations. First, because the effectiveness data were obtained from a cohort study, it may be subject to both selection bias and confounding commonly present in observational studies; and the COVID-19 pandemic may have further compromised study findings. For example, the cohort study used two measurements for smoking outcomes: a self-reported questionnaire and an exhaled carbon monoxide (CO) test as a biochemical validation method. The exhaled-CO test, however, was prohibited to prevent viral transmission. Additionally, the pandemic lead to reduced follow-up, resulting in decreased responses from study participants; among participants with CVD only 33% had complete outcome data. Our base case utilized an ITT analysis to obtain conservative estimates of the program efficacy and despite the use of these estimates in our model, the FAHSAI Clinic was found to be cost-effective. Further, there was a large difference in the 6-month CAR in the per-protocol and ITT analyses, 41.77% and 13.81%, respectively. Depending on available resources, each multidisciplinary FAHSAI Clinic may offer differing services and treatments. For example, only 68.40% of participants in their cohort were receiving any pharmacotherapies. As participants who had not used any pharmacotherapies had lower likelihoods of successfully quitting smoking, compared to those who had, it is evident that these variations in the services and treatments offered may have attenuated the program’s overall effectiveness. As such, we ran a scenario analysis using the per-protocol efficacy estimates and the FAHSAI clinic remained dominant over usual care over a 50-year time horizon, with less costs associated with gains in QALYs compared to the base-case analysis.

The second limitation was that because smoking cessation clinics are considered the standard care for Thai smokers, the current study compared the cost-effectiveness of FAHSAI Clinic to usual care but it was not possible to obtain the effectiveness of the clinic compared to self-quit attempt in analyses. There was also a lack of comparative evidence on subsequent quit attempts in participants receiving care at FAHSAI Clinics and under usual care. The model assumed that smokers do not attempt smoking cessation again after a first failed attempt until death, although several quit attempts may be required before they are successful. In addition, despite the possibility for smokers to develop multiple comorbidities, our model assumed that smokers would suffer from at most one smoking-related disease in their lifetime. However, this would likely underestimate the harm induced by smoking and result in an attenuation of the observed benefit from the smoking cessation interventions. Further, as we performed English-language searches, Asian-based data were limited and therefore the relative risks of death from smoking related disease and utility values used in our model were derived from studies done in the US, Europe, and Japan, which may reduce generalizability to Thai smokers.

### Considerations and implications

An important consideration was that this study considered multidisciplinary clinical smoking cessation as an intervention for tobacco control. To date, much of the research on smoking cessation interventions compare individual strategies or a combination of two strategy types. A systematic review and meta-analysis of randomized controlled trials, found that multiple behavioral change strategies were more effective than usual care and brief advice interventions alone^32^, however, the effectiveness rates reported were lower than those observed from the FAHSAI Clinic. In reality, population-wide policy, systems, and environmental changes have also been shown to be highly efficient and effective at reaching many people resulting in increased motivation to quit and demand for tobacco dependence treatment^33^. However, tailored programs such as the FAHSAI Clinic tend to be more effective compared with these standardized interventions, thereby providing greater effects on health behaviors^33^.

Our findings have policy implications to support smoking cessation services in Thailand. Despite being implemented in Thailand in 2010, the FAHSAI Clinic has not yet been included in the National Health Security Office (NHSO) health benefit package. Our results demonstrated its cost-effectiveness compared to usual care among Thai smokers with CVD, thereby providing strong support for policy makers to consider including FAHSAI as part of their benefit package.

## CONCLUSIONS

The FAHSAI Clinic was dominant as it was less costly and more effective compared to usual care over the 50-year time horizon. The most important factors that influenced the model were the incidence of stroke and CHD among males. The probability that FAHSAI Clinic was cost-effective was 99.8% at a willingness-to-pay value of $5120. Our findings have implications for clinicians and policymakers to support smoking cessation services for Thai smokers. A budget impact analysis is needed to estimate the financial impact of the adoption of this program within the Thai healthcare system.

## Data Availability

The data supporting this research are available from the authors on reasonable request.
