# Supplementary material for: Cost-effectiveness analysis of the SMART quit clinic program in smokers with cardiovascular disease in Thailand
Source: Tob Induc Dis. 2023 Apr 5;21:47. doi: 10.18332/tid/161024 (PMC10075194; doi:10.18332/tid/161024)
Supplement: Supplementary file 1 [file TID-21-47-s1.pdf]

SUPPLEMENTAL FIGURES AND TABLES

Supplemental Table 1. Transition probabilities applied for the coronary heart disease cohort health states

Patient	Tp1	Tp2	Tp3	Tp4	Tp5
**Current smokers**					
Males 35 to 64 years old	0.61%	3.9%	0.030%	0.026%	0.005%
Males 65 years old and above	3.11%	3.9%	0.065%	2.247%	0.041%
Females 35 to 64 years old	0.54%	3.9%	0.030%	0.019%	0.005%
Females 65 years old and above	3.22%	3.9%	0.247%	9.500%	0.024%
**Former smokers**					
Males 35 to 64 years old	0.37%	2.0%	0.030%	0.036%	0.005%
Males 65 years old and above	2.49%	2.0%	0.025%	1.623%	0.023%
Females 35 to 64 years old	0.24%	2.0%	0.030%	0.019%	0.005%
Females 65 years old and above	2.65%	2.0%	0.095%	6.874%	0.012%

Abbreviations: Tp- transition probability.

Supplemental Table 2. Transition probabilities applied for the stroke cohort health states

Patient	Tp1	Tp2	Tp3	Tp4	Tp5
**Current smokers**					
Males 35 to 64 years old	0.35%	0.81%	0.030%	0.026%	0.005%
Males 65 years old and above	0.93%	4.15%	0.065%	2.247%	0.041%
Females 35 to 64 years old	2.71%	0.72%	0.030%	0.019%	0.005%
Females 65 years old and above	0.89%	4.29%	0.247%	9.500%	0.024%
**Former smokers**					
Males 35 to 64 years old	0.15%	0.50%	0.030%	0.036%	0.005%
Males 65 years old and above	0.62%	3.32%	0.025%	1.623%	0.023%
Females 35 to 64 years old	1.07%	0.32%	0.030%	0.019%	0.005%
Females 65 years old and above	0.67%	3.53%	0.095%	6.874%	0.012%

Abbreviations: Tp- transition probability.

Supplemental Table 3. Key Markov model input parameters

Variable	Mean	Standard error	Distribution for probability sensitivity analysis	Reference/Source
Discounting	0.03	-		Sanders 2016
**Clinical parameters**				
**Smoking quit rates at 6 months**				
Usual care	0.05	0.02	Beta	Expert opinion
Smoking cessation clinic	0.14	0.06	Beta	Concurrent cohort study[1]
**Relapse rates post quitting**				
Up to 5 years	0.06	0.02	Beta	Wetter 2004
6 to 10 years	0.02	0.02	Beta	Krall 2002
11 years and over	0.01	0.02	Beta	Krall 2002
**Cost parameters (in USD)**				
Usual care	0	-	Gamma	Expert opinion
Smoking cessation clinic	60.16	-		
Cost of medication	56.96	-	Gamma	Concurrent cohort study[1]
Cost of general practitioner visits	3.20	-	Gamma	Concurrent cohort study[1]
**Acute CHD**				
First year	4252.63			
Direct medical cost	4141.94	633.97	Gamma	Anukoolsawat 2006, Choosakulchart 2013
Direct non-medical cost	110.70	16.94	Gamma	Anukoolsawat 2006, Choosakulchart 2013
Subsequent years	604.67			
Direct medical cost	444.57	68.05	Gamma	Anukoolsawat 2006, Choosakulchart 2013
Direct non-medical cost	160.10	24.51	Gamma	Anukoolsawat 2006, Choosakulchart 2013
**Stroke**				

Variable	Mean	Standard error	Distribution for probability sensitivity analysis	Reference/Source
First year	2882.33			
Direct medical cost	2189.70	335.16	Gamma	Tamteerano (Statin CEA by HITAP)
Direct non-medical cost	692.63	106.01	Gamma	Riewpaiboon 2010, Expert opinion
Subsequent years	531.60			
Direct medical cost	346.90	53.10	Gamma	Tamteerano (Statin CEA by HITAP)
Direct non-medical cost	184.70	28.27	Gamma	Riewpaiboon 2010, Expert opinion
**COPD**	2471.88			
Direct medical cost	2111.71	403.88	Gamma	Thanaviratananich 2016
Direct non-medical cost	360.17	55.13	Gamma	Riewpaiboon 2010, Expert opinion
**Oral cancer**	3521.64			
Direct medical cost	2782.84	425.94	Gamma	Weerapradits 2009
Direct non-medical cost	738.80	113.08	Gamma	Riewpaiboon 2010, Expert opinion
**Lung cancer**	6686.58			
Direct medical cost	6058.60	927.34	Gamma	Thongprasert 2017
Direct non-medical cost	627.98	96.12	Gamma	Riewpaiboon 2010, Data collection from Maharajnakorn Hosp, Chiang Mai
**Health utilities**				
**CHD**	0.76	0.02	Beta	Annemans 2009
**Stroke**				
First year	0.74	0.09	Beta	Annemans 2009
Subsequent years	0.15	0.02	Beta	Annemans 2009
**COPD**	0.76	0.1	Beta	Annemans 2009
**Oral cancer**	0.88	0.02	Beta	Downer 1997
**Lung cancer**	0.66	0.02	Beta	Chouaid 2013
**Incident morbidity risks - Incidence**				
**Current smokers**				
**CHD**				

Variable	Mean	Standard error	Distribution for probability sensitivity analysis	Reference/Source
Male (35-64)	0.61%	-	-	Friedman 2000
(65+)	3.11%	-	-	
Female (34-64)	0.54%	-	-	
(65+)	3.22%	-	-	
**Stroke**				
Male (35-64)	0.35%	-	-	Allender 2008
(65+)	0.93%	-	-	
Female (34-64)	2.71%	-	-	
(65+)	0.89%	-	-	
**COPD**				
Male (35-64)	0.03%	-	-	Allender 2008
(65+)	2.25%	-	-	
Female (34-64)	0.02%	-	-	
(65+)	9.50%	-	-	
**Lung cancer**				
Male (35-64)	0.03%	-	-	Thun 2000
(65+)	0.06%	-	-	
Female (34-64)	0.03%	-	-	
(65+)	0.25%	-	-	
**Oral cancer**				
Male (35-64)	0.005%	-	-	Ide 2008
(65+)	0.041%	-	-	
Female (34-64)	0.005%	-	-	
(65+)	0.024%	-	-	
**Former smokers**				
**CHD**				Friedman 2000
Male (35-64)	0.37%	-	-	
(65+)	2.49%	-	-	
Female (34-64)	0.24%	-	-	
(65+)	2.65%	-	-	
**Stroke**				Allender 2008
Male (35-64)	0.15%	-	-	
(65+)	0.62%	-	-	
Female (34-64)	1.07%	-	-	
(65+)	0.67%	-	-	

Variable	Mean	Standard error	Distribution for probability sensitivity analysis	Reference/Source
**COPD**				Allender 2008
Male (35-64)	0.03%	-	-	
(65+)	1.62%	-	-	
Female (34-64)	0.02%	-	-	
(65+)	6.87%	-	-	
**Lung cancer**				Thun 2000
Male (35-64)	0.03%	-	-	
(65+)	0.03%	-	-	
Female (34-64)	0.03%	-	-	
(65+)	0.09%	-	-	
**Oral cancer**				Ide 2008
Male (35-64)	0.005%	-	-	
(65+)	0.023%	-	-	
Female (34-64)	0.005%	-	-	
(65+)	0.012%	-	-	
**Incident mortality risks – Relative risks (Standard error)**				
**Current smokers**				
**CHD**				Friedman 2000
Male	2.0	0.04	-	
Female	2.2	0.04	-	
**Stroke**				Friedman 2000
Male (35-64)	2.4	0.13	-	
(65+)	1.5	0.10	-	
Female (34-64)	3.8	0.11	-	
(65+)	1.6	0.08	-	
**COPD**				Friedman 2000
Male	10.8	0.13	-	
Female	12.3	0.11	-	
**Lung cancer**				Ando 2003
Male	4.5	0.19	-	
Female	3.6	0.24	-	
**Oral cancer**				Ide 2008
Male	2.6	0.49	-	
Female	7.9	0.67	-	
**Former smokers**				

Variable	Mean	Standard error	Distribution for probability sensitivity analysis	Reference/Source
**CHD**				Friedman 2000
Male	1.0	0.07	-	
Female	1.5	0.05	-	
**Stroke**				Friedman 2000
Male (35-64)	1.0	0.14	-	
(65+)	1.0	0.07	-	
Female (34-64)	1.5	0.15	-	
(65+)	1.2	0.09	-	
**COPD**				Friedman 2000
Male	7.8	0.12	-	
Female	8.9	0.11	-	
**Lung cancer**				Ando 2003
Male	2.4	0.20	-	
Female	2.6	0.42	-	
**Oral cancer**				Ide 2008
Male	2.6	0.49	-	
Female	7.9	0.67	-	

[1] Real-World Assessment of the Thai Multidisciplinary Smoking Cessation Program on Clinical Outcomes: A Multicentre Prospective Observational Study

*COPD: Chronic obstructive pulmonary disease; CHD: Coronary heart disease

Supplemental Figure 1. Tornado diagram of one-way sensitivity analysis

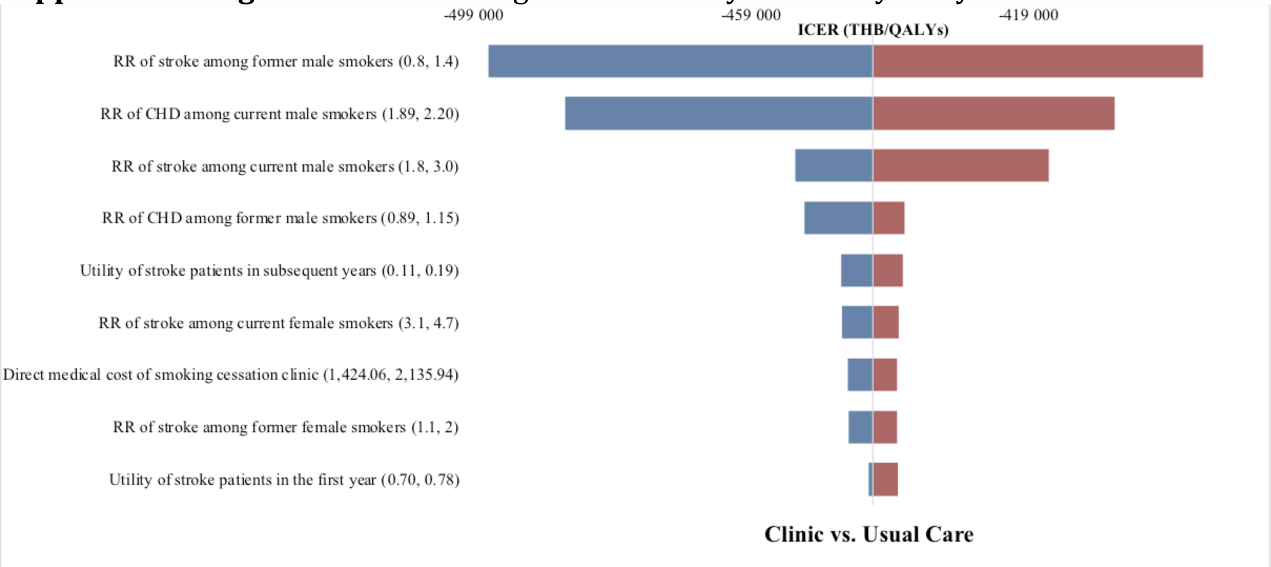

Abbreviations: CHD – coronary heart disease; RR- relative risk; ICER – incremental cost-effectiveness ratio; THB- Thai Baht; QALY- quality-adjusted life year; Clinic- The SMART Quit Clinic Program.

Supplemental Figure 2. Cost-effectiveness plane for the FAHSAI clinic in Thailand representing probability sensitivity analysis

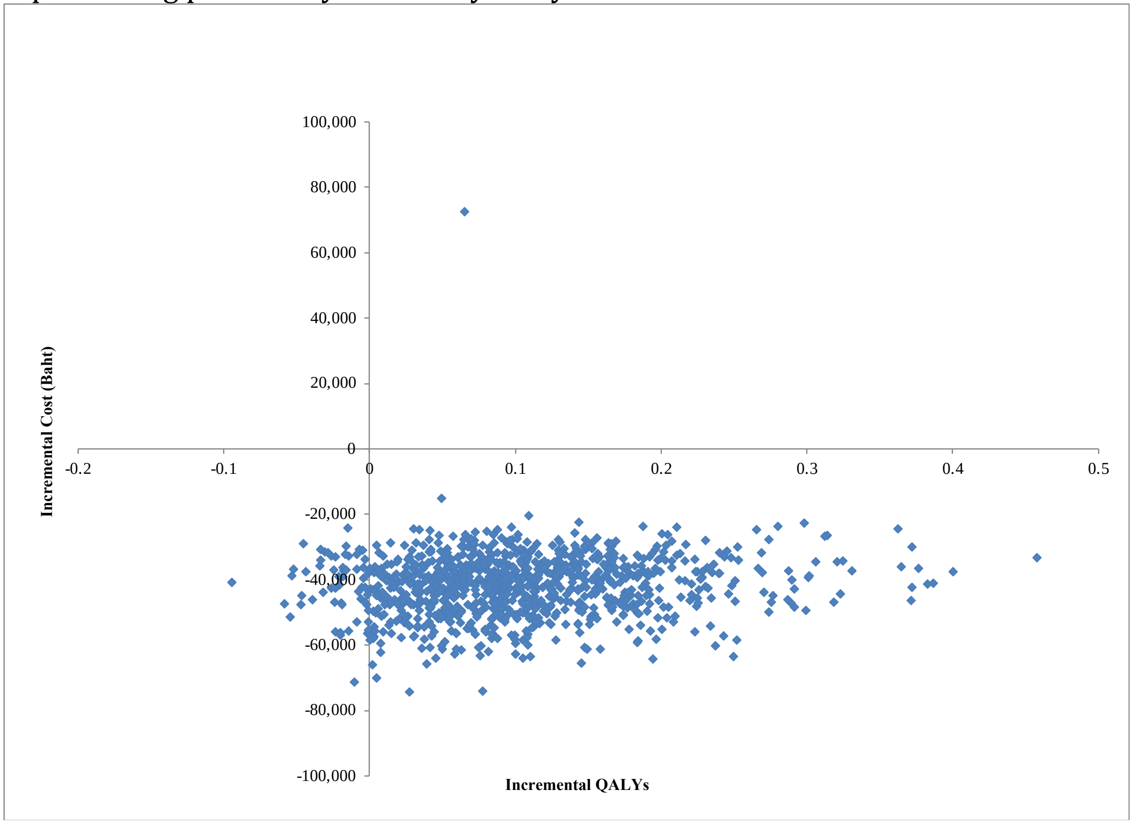

Abbreviations: QALY- quality-adjusted life year.

Supplemental Figure 3. Cost-effectiveness acceptability curve, FAHSAI clinic vs. usual care in Thailand

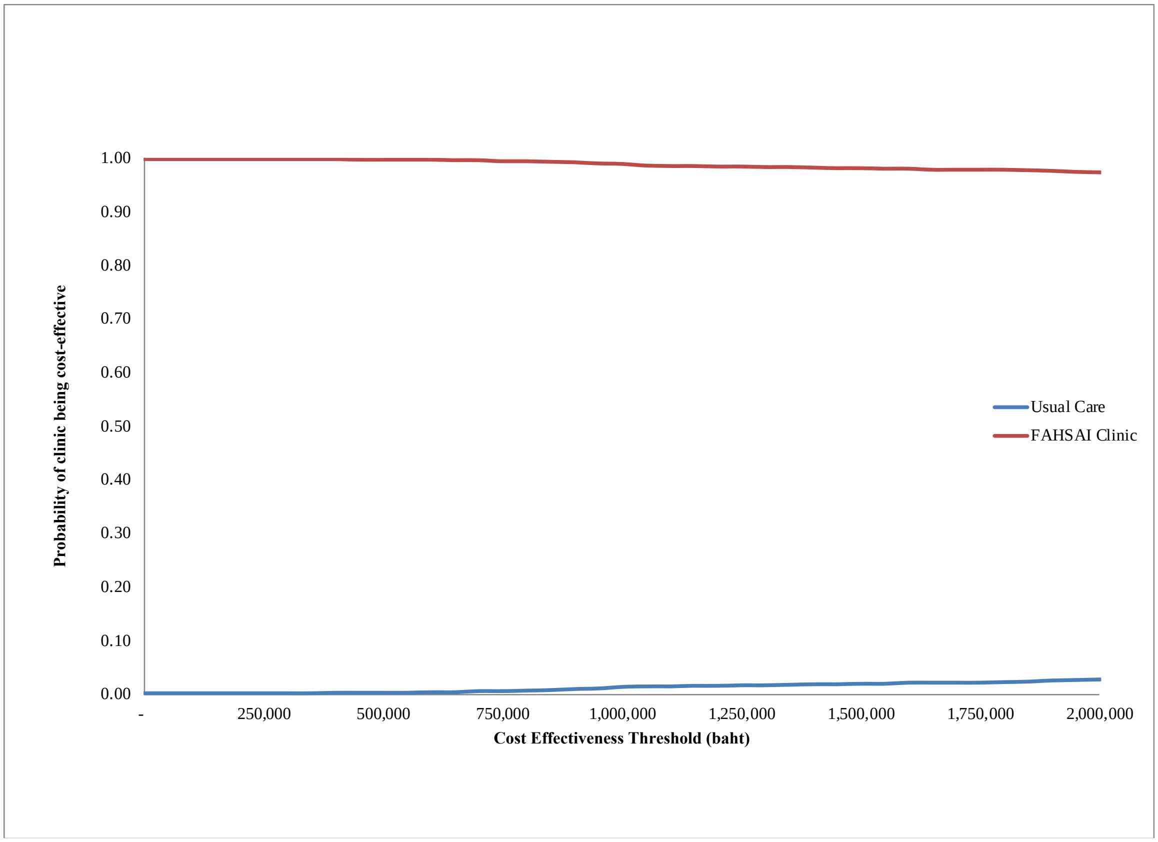

Abbreviations: FAHSAI Clinic- The SMART Quit Clinic Program.

Appendix 1. CHEERS 2022 Checklist

	Item	Reported in Section
**TITLE**		
Title	1	Title Page
**ABSTRACT**		
Abstract	2	Abstract
**INTRODUCTION**		
Background and objectives	3	Introduction
**METHODS**		
Health economic and analyses plan	4	2.2 - Model Structure
Study population	5	2.2 – Model Structure
Setting and location	6	2.2 – Model Structure
Comparators	7	2.2 – Model Structure
Perspective	8	2.1 – Overall Description
Time horizon	9	2.1 – Overall Description
Discount rate	10	2.1 – Overall Description
Selection of outcomes	11	2.3 – Input Parameters
Measurement of outcomes	12	2.3 – Input Parameters
Valuation of outcomes	13	2.3 – Input Parameters
Measurement and valuation of resources and costs	14	2.3 – Input Parameters
Currency, price date, and conversion	15	2.3 – Input Parameters
Rationale and description of model	16	2.1 – Overall Description
Analytics and assumptions	17	2.2 – Model Structure
Characterizing heterogeneity	18	2.4 – Sensitivity analyses
Characterizing distributional effects	19	2.4 – Sensitivity analyses
Characterizing uncertainty	20	2.4 – Sensitivity analyses
Approach to engagement with patients and others affected by the study	21	Discussion
**RESULTS**		
Study parameters	22	Results
Summary of main results	23	Results
Effect of uncertainty	24	Results
Effect of engagement with patients and others affected by the study	25	Results
**DISCUSSION**		
Study findings, limitations,	26	Discussion

generalizability and current knowledge		
**OTHER RELEVANT INFORMATION**		
Source of funding	27	Other relevant information
Conflicts of interest	28	Other relevant information

© 2023 Grant A. et al.
